# Effect of Chitosan and Naringin on Enteric Methane Emissions in Crossbred Heifers Fed Tropical Grass

**DOI:** 10.3390/ani11061599

**Published:** 2021-05-28

**Authors:** Rafael Jiménez-Ocampo, María Denisse Montoya-Flores, Esperanza Herrera-Torres, Gerardo Pámanes-Carrasco, Jeyder Israel Arceo-Castillo, Sara Stephanie Valencia-Salazar, Jacobo Arango, Carlos Fernando Aguilar-Pérez, Luis Ramírez-Avilés, Francisco Javier Solorio-Sánchez, Ángel Trinidad Piñeiro-Vázquez, Juan Carlos Ku-Vera

**Affiliations:** 1 Faculty of Veterinary Medicine and Animal Science, University of Yucatan, Merida C.P. 97100, Mexico; jeyder96@gmail.com (J.I.A.-C.); caperez@correo.uady.mx (C.F.A.-P.); luis.ramirez@correo.uady.mx (L.R.-A.); ssolorio@correo.uady.mx (F.J.S.-S.); 2 National Institute for Forestry, Agriculture and Livestock Research—INIFAP, Experimental Field Valle del Guadiana, Durango C.P. 34170, Mexico; 3 National Center for Disciplinary Research in Physiology and Animal Breeding, National Institute for Forestry, Crops, and Livestock Research—Ministry of Agriculture and Rural Development, Ajuchitlan C.P. 76280, Mexico; denis_montoya@yahoo.com.mx; 4 National Technologic of Mexico, Technological Institute of Valle del Guadiana, Durango C.P. 34371, Mexico; hetoes99@yahoo.com.mx; 5 Institute of Silviculture and Wood Industry, National Council of Science and Technology–Durango State Juarez University, Durango CP 34126, Mexico; gerardo.pamanes@gmail.com; 6 College of the Southern Border (ECOSUR), Livestock and Environment, San Cristobal de las Casas C.P. 29290, Mexico; saraudea@gmail.com; 7 International Center for Tropical Agriculture (CIAT), Palmira C.P. 763537, Colombia; j.arango@cgiar.org; 8 National Technologic of Mexico, Technological Institute of Conkal, Conkal C.P. 97345, Mexico; pineiroiamc@gmail.com

**Keywords:** additive, flavonoid, chitin, antimicrobial action, greenhouse gases

## Abstract

**Simple Summary:**

The increase in human population and the concomitant rise in demand for animal protein have contributed to augment enteric methane emissions. It is imperative to reduce methane, increase sustainable production, avoid the use of chemical compounds, and guarantee quality products for the consumer. Chitosan and naringin possess antimicrobial properties, and they have shown their capacity to reduce methane in in vitro trials. This study investigated their effects as feed additives given to improve ruminal fermentation and nutrient utilization and decrease methane in crossbred heifers fed tropical grass. In in vitro experiments, chitosan and naringin at three levels (0, 1.5, 3.0 g/kg) showed significant methane reductions when 1.5 g/kg of chitosan was included. The in situ study did not reveal changes in rumen degradability with the inclusion of the additives. However, in in vivo assays, chitosan and naringin at 1.5 or 3.0 g/kg dry matter intake or the combination of both compounds (1.5 and 1.5 g/kg) given directly into the rumen did not induce changes in rumen fermentation, methane production, or nutrient utilization. However, given the promising evidence from other studies, more research needs to be conducted to clarify the potential effects of chitosan and naringin in animal production.

**Abstract:**

In order to meet consumer needs, the livestock industry is increasingly seeking natural feed additives with the ability to improve the efficiency of nutrient utilization, alternatives to antibiotics, and mitigate methane emissions in ruminants. Chitosan (CHI) is a polysaccharide with antimicrobial capability against protozoa and Gram-positive and -negative bacteria, fungi, and yeasts while naringin (NA) is a flavonoid with antimicrobial and antioxidant properties. First, an in vitro gas production experiment was performed adding 0, 1.5, 3.0 g/kg of CHI and NA under a completely randomized design. The substrate containing forage and concentrate in a 70:30 ratio on a dry matter (DM) basis. Compounds increased the concentration of propionic acid, and a significant reduction in methane production was observed with the inclusion of CHI at 1.5 g/kg in in vitro experiments (*p* < 0.001). In a dry matter rumen degradability study for 96 h, there were no differences in potential and effective degradability. In the in vivo study, six crossbred heifers fitted with rumen cannulas were assigned to a 6 × 6 Latin square design according to the following treatments: control (CTL), no additive; chitosan (CHI1, 1.5 g/kg DMI); (CHI2, 3.0 g/kg DMI); naringin (NA1, 1.5 g/kg DMI); (NA2, 3.0 g/kg DMI) and a mixture of CHI and NA (1.5 + 1.5 g/kg DMI) given directly through the rumen cannula. Additives did not affect rumen fermentation (*p* > 0.05), DM intake and digestibility of (*p* > 0.05), and enteric methane emissions (*p* > 0.05). CHI at a concentration of 1.5 g/kg DM in in vitro experiments had a positive effect on fermentation pattern increasing propionate and reduced methane production. In contrast, in the in vivo studies, there was not a positive effect on rumen fermentation, nor in enteric methane production in crossbred heifers fed a basal ration of tropical grass.

## 1. Introduction

Ruminants are herbivores that maintain a symbiotic association with the gut microbiome that enables them to use fibrous/cellulosic materials and convert them into high-quality proteins (meat, milk) for human consumption [1]. Despite this, ruminant production is associated with the emissions of greenhouse gases (GHG) such as carbon dioxide (CO_2_), methane (CH_4_), and nitrous oxide (N_2_O), which implies considerable environmental costs [2,3,4]. In the process of anaerobic fermentation of carbohydrates in the rumen, methanogenic archaea utilize dihydrogen (H_2_) to reduce CO_2_ to CH_4_ as the end product of their metabolism [5]. Methanogenesis represents a loss of between 2% (grains, concentrates) to 12% (poor-quality forages) of gross energy intake (GEI) [6,7,8]. Therefore, scientific research has intensified to find natural antimicrobial compounds that may be used as feed additives without adverse effects on rumen fermentation patterns, are nontoxic to the animal, are economically viable to mitigate CH_4_ production, and can maintain or increase livestock productivity [9,10].

Chitosan is the collective name for a group of the partially or fully deacetylated biopolymer chitin, composed of two repeated units of D-glucosamine and N-acetyl-D-glucosamine, linked by β-(1⟶4)-linkages. It has antimicrobial properties against Gram-positive bacteria and, to a lesser extent, against Gram-negative bacteria, filamentous fungi, yeast, and even viruses [11,12]. The antimicrobial mechanism has not been fully described; it is assumed that it includes different mechanisms with electrostatic interactions or divalent cations, replacement of magnesium and calcium ions at the cell surface, destabilization of the cell membrane, inhibition of mRNA, and protein synthesis in cell nuclei, and death of microorganisms [13,14]. The antimicrobial capacity depends on the molecular weight, hydrophilicity, solubility, and degree of deacetylation of the parent CHI, pH (<6.5), pK_a_ (~6.3), and ionic strength in the medium [15]. Recent research on CHI has focused on its potential to modulate rumen fermentation in beef and dairy cattle [3,13,16,17].

On the other hand, naringin (NA; 4′,5,7-trihydroxyflavanone 7-rhamnoglucoside, C_27_H_32_O_14_, molecular weight 580.4 g/mol) is one of the 4000 flavonoids reported in the literature on citrus plants, which are a good source of these compounds that include NA, naringenin, nobelitin, narirutin, and hesperidin [18]. Naringin is responsible for the distinctive sour flavor and bitter taste of grapefruits and other citrus fruits. It possesses a wide range of biological effects such as antioxidant, anti-inflammatory (in vitro and in vivo), immune response, antiapoptotic, hepatoprotective, and cardioprotective effects, along with improving gut health and having a beneficial effect in certain metabolic diseases [19,20]. Flavonoids have antimicrobial properties that may participate in the reduction of methanogenic archaea, replace chemical compounds and, at the same time, improve animal health, feed efficiency, and productivity [21,22]. It has been recently proposed that the antimicrobial mechanisms of flavonoids function through the interference with specific intracellular or surface enzymes [21]. Relatively little information is available on the effect of chitosan and naringin in trials carried out in vivo in cattle. The rationale behind this trial was to assess the impact of supplementing CHI and NA on molar proportions of VFA in the rumen and enteric CH_4_ emissions of cattle fed a basal ration of tropical grass while kept in respiration chambers.

## 2. Materials and Methods

The experiments were carried out at the Laboratory of Climate Change and Livestock Production of the Faculty of Veterinary Medicine and Animal Science, the University of Yucatan in Merida, Mexico, located at 20°52′0.3″ N and 89°37′21″ W and 10 m above sea level. All experimental procedures with animals were carried out under the approval and following the guidelines and regulations for animal experimentation at the Campus of Biological and Agricultural Sciences (CCBA) of the University of Yucatan, Merida, Mexico.

### 2.1. In Vitro Experiment

#### 2.1.1. In Vitro Fermentation Patterns

For the in vitro gas production experiment, four cows (*Bos indicus* × *Bos taurus*) adapted to the diet for 15 days (70:30 forage:concentrate ratio) with a permanent rumen cannula (10 cm i.d., Bar Diamond Inc., Parma, ID, USA) served as donors of rumen liquor, which was obtained before the morning feeding (between 0800 and 0830 h) and subsequently transferred into a prewarmed insulated flask, previously filled with carbon dioxide, for laboratory analyses. The total mixed rations are shown in Table 1, and the inclusion of the two different compounds chitosan (Alfadelta, Estado de Mexico, Mexico; deacetylation degree 92.5%, viscosity 58 mPa.s) and naringin (RunYu BioTech, Baoji, China; 98.5% purity) according to the following treatments: (1) control treatment (CTL, no additive) (2) chitosan CHI1 (1.5 g/kg dry matter (DM); (3) chitosan CHI2 (3.0 g/kg DM); (4) naringin NA1 (1.5 g/kg DM); (5) naringin NA2 (3.0 g/kg DM), which were compared to a control treatment (without the compounds) under a completely randomized design. One gram of sample from each experimental treatment was incubated with 120 mL of ruminal inoculum buffer solution in a 2:1 ratio at 39 °C in ANKOM glass modules (ANKOM Technology, USA), equipped with hermetic rubber and plastic lids, as proposed by the manufacturer by triplicate (ANKOM 2018). After 24 h of incubation, the modules were opened, and pH was measured immediately; afterward, aliquots of 5 mL were taken for the analysis of volatile fatty acids (VFA) and ammonia nitrogen, as previously described by Araiza-Ponce [23].

#### 2.1.2. Gas and Methane Production

Approximately one gram of sample from rations of each experimental treatment was incubated by triplicate [24] and placed in ANKOM glass modules (ANKOM Technologies, Macedon, NY, USA), equipped with wireless pressure transducers. Fermentations were carried out according to the procedures proposed by the manufacturer [25] by incubating the sample with a mixture of a ruminal buffer inoculum solution in a 2:1 ratio. Incubations were performed from 0 to 96 h and the pressure was registered every hour. In vitro gas production kinetics was estimated by fitting data to the Gompertz [26] function according to the following equation:

GP = Gmax × exp (−A × exp (−k × t))(1)

where GP = gas production at time t (mL); Gmax = maximum gas production (mL); k = constant gas production rate (h^−1^); A = lag time before gas production begins (h). Once 24 h of incubation time had elapsed, for the measurements of the proportions of CH_4_ and CO_2_, the pressure relief valve of the modules was opened for 2 s, and the released gas was led through to a portable gas analyzer, according to the procedure proposed by the manufacturer (GEMTM5000, LANDTEC, Dexter, MI, USA) and adapted by González-Arreola [27].

#### 2.1.3. Statistical Analyses

All data were analyzed according to a completely randomized design, using the GLM procedure of the statistical package SAS^®^ 9 (SAS Inc., Cary, NC, USA) [28]. The model employed was as follows:

Y = μ + Ti + e(2)

where μ is the overall mean, Ti is the treatment effect, and e is the error term. Standard error of the difference among means was carried out using least square means. The comparison of means was carried out using the Tukey test, declaring significant differences at *p* < 0.05. Orthogonal contrast analyses were performed to evaluate the linear and quadratic effect of CHI treatments.

### 2.2. In Vivo Experiment

#### 2.2.1. Animals, Experimental Design, and Treatments

Six crossbred heifers (*Bos indicus* × *Bos taurus*) fitted with rumen cannulas (10 cm i.d.; Bar Diamond Inc. Parma, ID, USA) with an average live weight (LW) of 360 ± 12 kg were used. Heifers were randomly assigned to a 6 × 6 crossover Latin square design with periods of 21 days; 17 d for adaptation and 4 d for data collection. Treatments evaluated were (1) control (CTL, no additive); (2) chitosan CHI1 (1.5 g/kg DMI); (3) chitosan CHI2 (3.0 g/kg DMI), which presented the following technical specifications: deacetylation degree 92.5%, viscosity 58 mPa.s (Alfadelta, Estado de Mexico, Mexico); (4) naringin NA1 (1.5 g/kg DMI); (5) naringin NA2 (3.0 g/kg DMI), (98.5% purity, RunYu BioTech, Baoji, China); (6) CHI and NA (CHI–NA: 1.5 and 1.5 g/kg DMI, respectively). Heifers were housed in individual pens with free access to water. Before the experiment, cattle were dewormed with Doramectin 1 mL/50 kg LW (Dectomax^®^, Guadalajara, Mexico), and ADE vitamins (Vigantol ADE^®^, Bayer, Köln, Germany) were applied intramuscularly (1 mL/10 kg LW). Heifers were weighed at the beginning and the end of each period on a weighing scale (Vesta^®^, Santa Fe, Argentina).

#### 2.2.2. Experimental Ration, Feed Intake, and Apparent Digestibility

All heifers were fed the same total mixed ration (TMR), which covered the maintenance and growth requirements (11% crude protein (CP) and 9.0 MJ metabolizable energy (ME)/kg DM) according to the Nutrient Requirements of Beef Cattle [29]. The composition of the TMR is shown in Table 1, and it was offered at 2.8% of body weight; daily DMI was calculated to adjust the treatments. Heifers were fed the TMR once daily at 0800 h and the additives (CHI and NA) were supplied directly through the rumen cannula at the same time. Heifers had free access to water. Daily feed intake was calculated as the difference between the amount of feed offered, and that rejected the following day. Total production of feces [30] on days 17 to 21 of each period was collected for determining apparent digestibility, and 10% of the total was taken as the sample. For the manure collection, metabolic cages were fitted with a metal grid and a container for feces. Feed and feces samples taken daily were dried in a forced-air oven (55°C for 72 h) and ground through a 1 mm screen (Willey mill, Arthur H. Thomas Co., Philadelphia, PA, USA) for chemical analyses.

#### 2.2.3. In Situ Study

The same six cannulated heifers used in the in vivo experiment were used to estimate DM in situ degradation in periods 1, 3, and 5 [31]. At each incubation time, 5 g of feed TMR (Table 1) were weighed into nylon bags 10 × 20 cm; 50 ± 10-micron porosity per animal (R1020 Ankom Technology, Macedon, NY, USA) by duplicate inside the animal using the nylon bag technique [31]. The animal (*n* = 6) was considered the replicate of each treatment. Bags were incubated in the rumen at 0, 12, 24, 48, 72, and 96 h, in inverse order, to remove all bags simultaneously. After the incubation was completed, bags were manually washed under a running tap until the water from each bag was observed to be colorless and particle-free and then dried at 60 °C for 48 h.

In situ degradation curves (DM) were estimated with the exponential model described by Ørskov and McDonald [31] as follows:

P = a + b × (1 − exp^−ct^) (3)

where parameter a is the soluble and rapidly degradable fraction; parameter b is an insoluble but potentially degradable fraction; parameter c is fractional disappearance rate constant at which b is degraded; and P is the proportion (%) of dry matter degraded at time t (hours of incubation); the potential degradability values (PD, %) were estimated as the sum of a + b; effective degradability (ED) was calculated using the following equation:

ED = a + ((b × c)/(c + k))(4)

where a, b and c are the same parameters as described earlier, and k is the estimated rate of passage of 0.05/h for ruminants fed at low levels of production [29].

#### 2.2.4. Ruminal Fermentation Parameters

Rumen pH and proportions of VFA were measured in samples of ruminal liquor taken 5 h after feeding. On day 17 of each period, 50 mL of rumen fluid was collected through the rumen cannula using a syringe attached to a stainless-steel tube (20 mm internal diameter) from the anterior dorsal, anterior ventral, medium ventral, posterior dorsal, and posterior ventral locations in the rumen. A subsample of 4 mL of ruminal liquor was placed into a plastic tube containing 1 mL of metaphosphoric acid at 25% and stored at −20°C for volatile fatty acids (VFA) determination by gas chromatography [32]. Rumen pH was measured immediately after sampling with a portable pH meter (Hannah^®^ Instruments, Woonsocket, RI, USA). pH, which is the logarithm of the reciprocal of the hydrogen ion concentration in gram moles per liter, was transformed for analysis.

#### 2.2.5. Methane Production

Methane production was measured during three consecutive days (days 19 to 21 of each period) for 23 h per day using an infrared CH_4_ analyzer (MA-10 Sable Systems International^®^, Las Vegas, NV, USA) connected to two open-circuit respiration chambers with a volume of 9.3 m^3^ [33]. Temperature and relative humidity inside the chambers were kept at 23 °C and 55%, respectively. The air inside the chamber was extracted by two mass flowmeters (Sable Systems International^®^, Las Vegas, NV, USA) at a rate of one liter per kilogram LW/minute, as described by Valencia-Salazar [34]. The infrared CH_4_ analyzer was calibrated before each experimental period by injecting CH_4_ from a cylinder with a concentration of 1000 ppm CH_4_ diluted in nitrogen. Liters of methane produced per day were transformed into grams. Energy loss as CH_4_ was calculated from the heat combustion of CH_4_ = 55.65 MJ/kg^−1^ [35].

#### 2.2.6. Chemical Analysis

Dry matter contents of the diet, refusals, and feces were determined by drying samples in a forced-air oven at 105 °C for 48 h or until a constant weight was observed. Crude protein analysis was carried out on a LECO^®^ CN-2000 series 3740 instruments (LECO^®^ Corp., St. Joseph, MI, USA) (AOAC, method number 992.15). The ash content of the organic matter OM was determined by combustion in a muffle furnace at 550 °C (AOAC, method number 923.03). Ether extract (EE) was obtained by the Randall method (AOAC, method number 920.39). The concentration of neutral detergent fiber (NDF) (AN 3805 ANKOM, ANKOM Technology, Wayne County, NY, USA) and acid detergent fiber (ADF) were determined as suggested by Van Soest [36]. Gross energy (GE) was measured using an adiabatic bomb calorimeter (6400 Parr Instrument Company, Moline, IL, USA).

#### 2.2.7. Statistical Analyses

All data from in vivo experiment were subjected to analysis of variance for a 6 × 6 Latin square design, using the mixed procedure of the SAS^®^ 9.4 Software [28]. The statistical model was as follows:

Yijk = μ + Pi + Aj + Tk + Eijk(5)

where Y is the dependent variable, μ is the general mean, P is the effect of the period, A is the random effect of the animal, T is the effect of treatment, and E is the random residual error. Results were compared with the procedure LSmeans test, whereas orthogonal contrasts analysis were performed to evaluate the effect of CHI treatments.

Kinetics of degradation and potential rumen degradability were obtained from the equation proposed by Ørskov and McDonald [31] for each treatment using the nonlinear Marquardt procedure of SAS (version 9.1; SAS Inst., Inc., Cary, NC, USA), and the parameters of in situ degradation kinetics were analyzed with the mixed procedure of SAS where treatments were considered as fixed effects, and incubation replicates in the rumen were assumed to be a random effect. The model used for the analysis was as follows:

Yij = µ + Fi + Rj + eij(6)

where Y = the observation of the dependent variable ij; µ = the overall mean of Y; Fij = the effect of treatment (i = 6), R = the effect of incubation run as replicate (j = 4 animal); and eij = the random error associated with the observation ij. Standard error of the difference among means was carried out using least square means. Mean separation was performed using Tukey’s test, declaring significant differences at *p* < 0.05.

## 3. Results

### 3.1. In Vitro Experiments

#### 3.1.1. In Vitro Fermentation Patterns

The pH was not affected (*p* > 0.05) by the inclusion of CHI or NA (Table 2). At the same time, the compounds under evaluation modified ammonia nitrogen production and molar proportions of volatile fatty acids by increasing propionic acid and reducing acetic acid (*p* < 0.05). 

#### 3.1.2. Gas and Methane Production

There were no significant differences for total gas production and gas production kinetic parameters (*p* > 0.05); however, for methane production, CHI1 showed a reduction of 31.7% (*p* > 0.05) when compared to control (CTL); in contrast, CHI2 presented a higher (*p* > 0.05) methane production when compared to CTL (Table 3). The addition of NA did not affect methane production when compared to control (*p* > 0.05).

### 3.2. In Vivo Experiments

#### 3.2.1. Feed Intake and Apparent Digestibility

Ration supplementation of CHI and NA did not affect DMI, OM intake, CP intake, NDF intake, ADF intake (kg/day), nor GE intake (MJ/day) (*p* > 0.05) (Table 4). Similarly, ration digestibility was not affected by the inclusion of the additives (*p* > 0.05), except for CP digestibility (*p* < 0.01). Lower apparent digestibility of CP for CHI1 (80.18 g kg^−1^ DMI), CHI2 (81.35 g kg^−1^ DMI), and NA1 (82.52 g kg^−1^ DMI) treatments was observed, and the highest digestibility was obtained with the control treatment (87.13 g kg^−1^ DMI) (*p* < 0.05). Moreover, NA1 and CHI1 reduced the apparent digestibility of ADF (*p* = 0.01).

#### 3.2.2. In Situ Study

There were no differences in PD and ED at the different inclusions levels of the additives (Table 5).

#### 3.2.3. Rumen Fermentation Parameters

Rumen pH (average: 6.6) was not affected by CHI or NA inclusion (*p* > 0.05) (Table 6). Molar proportions of VFA in the rumen and the acetate:propionate ratio was not affected (*p* > 0.05) by the intraruminal administration of CHI or NA in the rations. Propionate and acetate molar proportions across treatments had an average of 24.57 and 48.87%, respectively, and the acetic:propionic acid ratio averaged 1.99.

#### 3.2.4. Methane Production

CHI and/or NA did not induce any effect on enteric CH_4_ production expressed as g day^−1^, g CH_4_ per kg DMI or digestible fractions, energy loss as CH_4_, Ym, and EF (*p* > 0.05) (Table 7) [35]. Energy loss as CH_4_ ranged between 8.52 to 9.35 MJ GE day^−1^, while Ym ranged from 6.95 to 7.30, and the EF ranged from 55.95 to 61.38 kg CH_4_/animal/year.

## 4. Discussion

### 4.1. In Vitro Experiments

In agreement with previous reports, pH was not affected (*p* > 0.05) by the addition of CHI and NA [22,37,38,39]. As in previous trials, chitosan addition increased the concentration of propionate and reduced that of acetate [3,40]. Jin-Jin [38] reported that the use of chitosan (3000 molecular weight) could change fermentation pathways, increasing propionate and amylolytic bacteria. As regards NA, Olagaray et al. [41] showed an increase in propionate concentration relative to acetate as in the present study, and it is known that flavonoids are beneficial in balancing rumen pH in subacute acidosis. Naringin at 4.5% (*w*/*w*), as with other flavonoids, reduced methane production without negative effects on rumen microbial fermentation [22]; the amount we used in the present study may have been low, but the inclusion of higher amounts may become an expensive issue under practical farming conditions. Gas production was not affected by the inclusion of the compounds, and this may suggest that there are no modifications in the fermentative microbial activity [42]. As regards CHI, previous studies reported a reduction of gas production using chitin and chitosan and a decrease in the digestibilities of organic matter and dry matter of the ration; the latter was probably due to its antiprotozoal effect [40]. In a meta-analysis, in vitro gas production showed that an increase in the doses of chitosan was associated with a decrease in total gas production (*p* < 0.001), reduction of the protozoa population (*p* < 0.05), and an increase of the total microbial population (*p* < 0.01) [43].

Other possibilities are lower H_2_ concentration, a shift in the bacterial community, or an antimicrobial effect against methanogens. However, the inclusion of the additive at a dose of CHI of 3.0 g/kg showed a negative effect, increasing methane production. In the NA case, there is little scientific evidence on flavonoids and NA action on the rumen microbial fermentation; some reports associate this reduction with changes in the ciliated protozoal community [37]. Furthermore, it must be considered that in vitro experiments have some limitations such as no absorption of VFA against time, passage rate variations, changes in the structure of the microbiome, and volume of reactions [44].

### 4.2. In Vivo Experiment

#### 4.2.1. Feed Intake and Apparent Digestibility

Dry matter intake, with the inclusion of CHI in the ration, agrees with results reported by Araújo et al. [16]. However, these differ from other reports [14,45,46] insofar as that in the present trial, the inclusion of CHI reduced apparent digestibility of CP and ADF, contrasting with the results obtained by Araujo [16] and Mingoti [45] using levels of 100 and 150 mg/kg of body weight. This suggests that the protein digestibility decreases as a result of the antimicrobial action of CHI on rumen bacteria and the defaunation of protozoa. At the same time, the reduction in ADF digestibility may be explained by adverse effects on ruminal fermentative processes, reducing the activity of cellulolytic bacteria or activity of fibrolytic enzymes, as has been observed with the inclusion of some secondary plant metabolites [3,47,48,49]

Naringin addition or the mixture (CHI:NA) did not affect DMI and apparent digestibility, which partially agrees with results using extracts of turmeric flavonoids, grape seeds, and green tea, which showed that dry matter intake was not increased [41]. Feed intake was not modified in the present trial, given the small amounts of the components (CHI and/or NA) added. Since the additives were introduced directly through the rumen cannula, the flavor and/or filling effect of the diet were not affected by the components used, and this was probably the reason why there were no changes in rumen fermentation.

#### 4.2.2. In Situ Study

CHI and NA showed no adverse effects on potential and effective rumen degradabilities of DM (*p* > 0.05) in the fermentation kinetics, of which only a few studies have been performed to date [50]. The constant rate of degradation (c), the effective degradability of dry matter (ED), and the soluble A and insoluble B fractions were not affected by the inclusion of the additives, which suggests that perhaps the compounds did not interact with the ingredients of the diet. One of the previous studies carried out by Goiri [51] suggested that CHI in in vitro experiments decreases the rumen degradation rate of fibrous feeds by the effect on cellulolytic bacteria, which was not the case in the present study with the concentrations evaluated.

#### 4.2.3. Rumen Fermentation Parameters

In previous reports, CHI supplementation increased the molar proportion of propionic acid (7%) and decreased the acetate:propionate ratio with the inclusion of 150 mg/kg BW in beef cattle [16] and in sheep using a dose of 136 mg/kg BW supplied through the rumen cannula [51]. These changes have been attributed to the notion that chitosan affects Gram-positive bacteria [52]. In contrast, there was no effect of CHI levels on rumen pH, VFA, and the C3:C2 (propionate:acetate ratio) in the present study. Changes in fermentation reported in previous trials did not occur in the present study, probably because the discrete amounts administered were not enough to modify the bacterial community (Bacteroidetes and Proteobacteria); for example, 4 g/kg DM of CHI in multiparous Holstein cows [53,54] or diets used were different from that used in the present study (70:30 forage:concentrate ratio) vs. other reports [14,16,46,55]. Rumen pH values were not modified, maybe because of the low grain (starch) included in rations and the rumen buffering activity, and therefore, populations of fibrolytic bacteria were not affected. Hence, the apparent digestibility of NDF showed no effect, which agrees with previous experiments in buffaloes [56].

Chitosan did not modify rumen fermentation between treatments. This may be related to factors such as rumen pH, given that the CHI molecule becomes polycationic when pH is below 6.5 and pK_a_ ~6.3, which promotes its antimicrobial capacity, along with its molecular weight [16,38,57,58]. In the present study, rumen pH was 6.60 and 6.53 for CHI1 and CHI2, respectively. Hence, no changes in microbial communities were expected. This is in accordance with the results on VFA proportions and CH_4_ production. However, VFA values found in all the treatments, including CTL, were consistent with diets that contain high amounts of grains (starch); consequently, a low pH will induce a low methane production and a reduction in NDF digestion [59]. Other factors involved for which no modification was possibly recorded are diet, forage quality, forage:concentrate ratio, and NDF intake [60].

Previous reports on flavonoids have shown that degradation products including NA may be metabolized to volatile fatty acids and thus modify microbial diversity in the rumen, enhancing the molar proportion of propionate while decreasing the acetate:propionate ratio [19,61]. Balcells et al. [62] fed a concentrate, barley straw, and supplemented Fleckvieh heifers with Bioflavex (containing NA) (0.3 g/kg DM) and recorded an increase in the molar proportion of propionate. Other authors did not observe differences in fermentation patterns in in vitro experiments [22]. Likewise, NA did not modify rumen pH in the present study, which is consistent with in vitro results by Oskoueian et al. [22] and Kim et al. [37]. These reports have shown that flavonoids and commercial flavonoid products promote favorable conditions for lactate-consuming microorganisms, thus preventing acidosis [63].

#### 4.2.4. Methane Production

Regarding methane production, Henry et al. [13] evaluated doses of 5 and 10 g/kg DM of CHI. They found no effect on enteric CH_4_ emissions in beef heifers regardless of the type of ration, either low or high in concentrate. It is possible that the doses used in the current experiment (1.5 and 3.0 g/kg DM) and rumen pH were not sufficient to induce changes in the population of methanogenic archaea, as antimicrobial activity of CHI is enhanced at low pH values [17]. On the other hand, biological activities of chitosan are compromised by its structural characteristics, including the degree of deacetylation, molecular weight, modification of structure, and types of linking depending on extraction origin [38,64,65].

The bactericidal capacity of flavonoids, including NA, has been described, and their ability to reduce methane production (by affecting protozoa and methanogens) has been described in in vitro experiments [22,63]. Previous reports carried out in vivo described modulation of the activity of rumen microbiota by increasing lactate-consuming bacteria as well as modulating rumen fermentation resulting in a higher molar proportion of propionate [66]. We did not find a reduction in rumen methane production when NA was supplied, probably due to the low concentration of NA in the rumen, which seems to agree with previous results with other flavonoids such as quercetin [67]. As far as the authors are concerned, there have been no previous studies evaluating NA to mitigate enteric methane under in vivo conditions with cattle housed in respiration chambers. There is a wide discrepancy in the literature on the results of the effect of flavonoids on methanogenesis, and more in vivo studies are needed to fully understand their mode of action in in vivo assays and how the type of diet (forages vs. grains) affects the response. It is also highly recommendable to give a closer look at the possible effects of CHI and NA on the composition of the rumen microbial population, particularly on the archaea.

Although no effect of CHI and NA was recorded in the experiment hereby described, these results set the basis from which new approaches can be envisioned in the effort to use those compounds as methane-mitigating agents, most likely by increasing the doses employed or by verifying possible changes in the microbiome or way of administration in forthcoming trials.

### 4.3. In Vitro vs. In Vivo Experiments

Although the in vitro gas production techniques are widely used for the evaluation of methane production, it is important to carry out in vivo evaluations even when they are labor intensive, time consuming, and expensive [68,69]. The discrepancy in the present results obtained between in vitro vs. in vivo trials could be related to the fact that the in vitro experiments do not consider the complex process of ruminal fermentation, rumen pH, the pattern of volatile fatty acids, absorption of VFA, passage rate, rumen dilution, the volume of the rumen, hindgut fermentation, and alterations in microbiome structure [70,71]. In the in vivo experiment, the additives (CHI, NA) by the intraruminal administration possibly did not allow a uniform distribution of the compounds in the rumen sacs (dorsal, ventral, blind) of approximately 200 L capacity in adult cows [44]; perhaps the product only had a slight effect on a subpopulation of microorganisms associated with the liquid phase but not on the microorganisms attached to the substrate or attached to the rumen wall [70]. Additionally, direct administration of the additives into the rumen affects the time of exposure of compounds. Perhaps future experiments may consider the administration in feed or an excipient for its administration (gel beads and alginate matrices). Another possibility is to search for a methane-reducing effect by using higher concentrations and different ration components, avoiding negative effects in terms of productivity and cost effectiveness.

## 5. Conclusions

Based on the results hereby presented, it can be concluded that in the in vitro studies, CHI at 1.5 g/kg DM and NA 3.0 g/kg DM had a positive effect on fermentation patterns, increasing propionic acid while reducing acetate and methane production by 12% and 31%, respectively. Both CHI and NA showed no effect on the kinetics of rumen DM degradation of the basal ration. For the in vivo trial where CHI and NA were administered either separately (1.5 or 3.0 g/kg DMI) or in a combination of NA and CHI (1.5 and 1.5 g/kg DMI, respectively) given directly into the rumen, both additives did not show a positive effect on rumen fermentation or enteric methane production. In spite of the lack of effect of the additives under in vivo conditions, as shown in the present trial, they present various biological potentialities that require further investigation. In the case of chitosan, these include the degree of deacetylation, molecular weight, modification of structure, and types of chemical bonds, while for naringin, they include the optimal type and dose in production rations to reduce methane emissions and the interaction between ingredients and nutrients on rumen function. The cost of this mitigation strategy must also be carefully considered under practical field conditions.

## Figures and Tables

**Table 1:** Ingredients and composition of the total mixed ration (TMR).

**Ingredient Inclusion (g/kg DM)**	**TMR**
Guinea grass hay *(Megathyrsus maximus)* ^1^	700
Ground corn	180
Soybean meal	85
Urea	30
Minerals	5
**Item (g/kg DM)**	
Dry matter	942 ± 1.52
Organic matter	928 ± 1.04
Crude protein	110 ± 1.15
Ether extract	7.0 ± 0.25
Ash	72 ± 1.56
Neutral detergent fiber	706 ± 1.52
Acid detergent fiber	393 ± 1.70
Gross energy (MJ/kg DM)	14.6 ± 0.26

^1^ Nutrient composition of Guinea grass hay (g/kg DM): DM 909.8; CP: 92.10; Ash 69.9; Ether extract 7.5.

**Table 2:** In vitro ruminal fermentation patterns.

Item	Treatments					SEM	*p*-Value	Contrast	
	CTL	NA1	NA2	CHI1	CHI2			L	Q
pH ^1^	6.8 ± 2.9E−8	6.9 ± 4.1E−8	6.8 ± 5.2E−8	6.8 ± 5.2E−8	6.7 ± 3.7E−8	1.8E−8	0.387	0.524	0.626
N-NH_3_ (mg/dL)	13.7 ± 0.07 ^c^	17.8 ± 0.16 ^a^	15.7 ± 0.16 ^b^	17.2 ± 0.35 ^a^	12.8 ± 0.19 ^c^	0.210	<0.001	<0.001	0.055
**Molar proportions of VFA (%)**									
Acetic	46.6 ± 0.01^a^	40.9 ± 0.65 ^b^	40.7 ± 0.36 ^b^	41.9 ± 0.99 ^b^	43.1 ± 0.11 ^b^	0.555	0.003	<0.001	0.004
Propionic	18.6 ± 0.63 ^c^	24.0 ± 0.01 ^b^	25.9 ± 0.16 ^a,b^	24.8 ± 0.32 ^a,b^	26.2 ± 0.23 ^a^	0.340	<0.001	<0.001	<0.001
Isobutyric	5.0 ± 0.59	4.4 ± 0.67	4.6 ± 0.11	3.6 ± 0.11	3.5 ± 0.10	0.411	0.18	0.994	0.043
Butyric	18.6 ± 0.16 ^b,c^	19.9 ± 0.37 ^a,b^	18.3 ± 0.23 ^c^	20.0 ± 0.22 ^a^	18.6 ± 0.01 ^b,c^	0.231	0.009	0.370	0.014
Isovaleric	7.9 ± 0.15 ^a^	7.3 ± 0.17 ^a^	7.3 ± 0.17 ^a^	6.3 ± 0.13 ^b^	5.8 ± 0.00 ^b^	0.141	<0.001	0.788	<0.001
Valeric	2.5 ± 0.08 ^a,b^	3.3 ± 0.15 ^a^	2.9 ± 0.01 ^a,b^	3.1 ± 0.21 ^a,b^	2.6 ± 0.02 ^b^	0.120	0.042	0.842	0.415
Acetic:propionic acid ratio	2.5 ± 0.08 ^a^	1.7 ± 0.02 ^b^	1.5 ± 0.00 ^b^	1.6 ± 0.06 ^b^	1.6 ± 0.01 ^b^	0.049	<0.001	<0.001	<0.001

CTL, control treatment; NA1, naringin (1.5 g/kg DMI); NA2, naringin (3.0 g/kg DMI); CHI1, chitosan (1.5 g/kg DMI); CHI2, chitosan, (3.0 g/kg DMI). ^1^ Standard error of pH is expressed in H ions. ^abc^ Different letters in the same row are statistically different; SEM, standard error of the difference among means; surface response: L: linear contrast; Q: quadratic contrast. VFA, volatile fatty acids.

**Table 3:** Effect of addition of naringin and chitosan on in vitro methane production.

Item	Treatments					SEM	*p*-Value	Contrast	
	CTL	NA1	NA2	CHI1	CHI2			L	Q
Gmax (mL/g DM)	602	607.5	594.3	504.3	572.0	29.49	0.16	0.36	0.24
A (h)	2.5	2.7	2.8	2.8	2.8	0.11	0.42	0.21	0.22
K(%/h)	0.07	0.09	0.08	0.09	0.10	0.008	0.30	0.41	0.06
GP24 (mL/g DM)	417.5	436.6	413.3	405.0	431.6	17.90	0.71	0.68	0.53
Methane (mL/g DM)	40.9 ^b^	43.2 ^b^	35.9 ^b,c^	27.9 ^c^	51.8 ^a^	1.78	<0.001	0.36	0.01

CTL, control treatment; NA1, naringin (1.5 g/kg DM); NA2, naringin (3.0 g/kg DM); CHI1, chitosan (1.5 g/kg DM); CHI2, chitosan, (3.0 g/kg DM). Gmax, maximum gas production; A, lag period before the gas production begins (lag phase); K, constant gas production rate; GP24, gas production at 24 h of fermentation time. ^abc^ Different letters in the same row are statistically different; SEM, standard error of the difference among means; surface response: L: linear contrast; Q: quadratic contrast.

**Table 4:** Effect of naringin and chitosan supplementation at different levels on feed intake and digestibility in heifers.

Item	Treatments						SEM	*p*-Value	Contrast	
	CTL	NA1	NA2	CHI1	CHI2	CHI–NA			L	Q
**Intake (kg/day)**										
DM	9.04 ± 0.46	8.84 ± 0.89	8.73 ± 0.37	8.69 ± 0.65	8.97 ± 0.90	8.93 ± 0.94	0.64	0.93	0.41	0.66
OM	7.43 ± 0.38	7.27 ± 0.73	7.17 ± 0.24	7.15 ± 0.57	7.38 ± 0.79	7.40 ± 0.75	0.53	0.94	0.41	0.73
CP	0.99 ± 0.05	0.97 ± 0.98	0.96 ± 0.41	0.95 ± 0.71	0.99 ± 0.10	0.98 ± 0.10	0.07	0.93	0.41	0.66
NDF	6.38 ± 0.32	6.24 ± 0.63	6.16 ± 0.26	6.13 ± 0.46	6.33 ± 0.63	6.30 ± 0.66	0.45	0.92	0.41	0.66
ADF	3.55 ± 0.18	3.47 ± 0.35	3.43 ± 0.15	3.41 ± 0.25	3.52 ± 0.35	3.51 ± 0.37	0.25	0.92	0.41	0.66
GE (MJ/day)	130.7 ± 6.62	127.8 ± 12.8	126.3 ± 5.38	125.7 ± 9.39	129.7 ± 13.0	129.1 ± 13.5	9.25	0.92	0.41	0.66
**Apparent digestibility (%)**										
DM%	65.8 ± 2.43	62.7 ± 1.92	64.3 ± 2.19	61.8 ± 1.76	64.1 ± 1.91	63.4 ± 2.13	1.63	0.43	0.20	0.39
OM%	62.7 ± 2.64	59.6 ± 2.28	61.4 ± 2.66	58.2 ± 1.94	61.1 ± 2.20	60.41 ± 2.77	1.83	0.45	0.25	0.39
CP%	78.1 ± 1.55 ^a^	73.5 ± 1.50 ^b,c^	75.7 ± 1.67 ^a,b^	70.6 ± 1.36 ^c^	72.3 ± 1.57 ^c^	75.1 ± 1.74 ^a,b^	1.19	0.0009	0.014	0.0013
NDF%	64.7 ± 2.50	60.0 ± 2.25	63.5 ± 2.52	59.2 ± 1.88	62.6 ± 2.11	63.4 ± 2.56	1.74	0.11	0.05	0.50
ADF%	60.7 ± 2.78 ^a^	53.7 ± 2.60 ^b^	60.5 ± 2.57^a^	53.0 ± 2.18 ^b^	58.2 ± 2.36 ^ab^	58.7 ± 2.89 ^ab^	1.94	0.014	0.02	0.61

CTL, control treatment; NA1, naringin (1.5 g/kg DMI); NA2, naringin (3.0 g/kg DMI); CHI1, chitosan (1.5 g/kg DMI); CHI2, chitosan, (3.0 g/kg DMI); and mixture of CHI and NA (CHI-NA, 1.5 + 1.5 g/kg DMI). DM, dry matter; OM, organic matter; CP, crude protein; NDF, neutral detergent fiber; ADF, acid detergent fiber; GE, gross energy means in the same row with different superscript letters differ (*p* < 0.05); SEM, standard error; surface response: L, linear contrast; Q, quadratic contrast.

**Table 5:** In situ rumen degradation kinetics, potential degradability, and effective degradability of treatments.

Item	Treatments						SEM	*p*-Value
	CTL	NA1	NA2	CHI1	CHI2	CHI–NA		
a (%)	28.75 ± 2.77	28.38 ± 2.26	27.73 ± 1.67	27.44 ± 1.91	28.09 ± 1.56	29.12 ± 1.54	2.00	0.98
b (%)	40.89 ± 6.05	38.37 ± 2.22	40.08 ± 1.56	39.04 ± 1.37	39.69 ± 0.97	38.50 ± 2.69	3.00	0.99
c (h^−1^)	0.04 ± 0.009	0.04 ± 0.006	0.04 ± 0.004	0.04 ± 0.006	0.04 ± 0.007	0.04 ± 0.008	0.01	0.98
PD (%)	69.65 ± 3.39	66.75 ± 0.55	67.81 ± 1.89	66.48 ± 0.93	67.78 ± 1.70	67.62 ± 2.40	2.04	0.87
ED (%)	45.62 ± 3.33	45.65 ± 2.49	44.65 ± 2.11	43.63 ± 2.92	45.75 ± 1.88	45.86 ± 1.57	2.46	0.97

a, soluble fraction; b, insoluble but potentially degradable fraction; c, fractional disappearance rate constant at which b is degraded; potential degradability (PD) and effective degradability (ED, considering a passage rate of 5% per hour); CTL, control treatment; NA1, naringin (1.5 g/kg DM); NA2, naringin (3.0 g/kg DM); CHI1, chitosan (1.5 g/kg DM); CHI2, chitosan, (3.0 g/kg DM) and mixture of CHI and NA (CHI-NA, 1.5 + 1.5 g/kg DM). SEM, standard error of the mean.

**Table 6:** Effect of naringin and chitosan supplementation on rumen fermentation parameter in heifers.

Item	Treatments						SEM	*p*-Value	Contrast	
	CTL	NA1	NA2	CHI1	CHI2	CHI–NA			L	Q
pH ^1^	6.6 ± 1.5E−7	6.3 ± 7.4E−7	6.7 ± 6.4E−8	6.5 ± 2.5E−7	6.4 ± 3.2E−7	6.5 ± 9.2E−8	3.0E−6	0.69	0.45	0.72
**Volatile fatty acids (molar%)**										
Acetate	49.27 ± 0.30	48.54 ± 0.33	48.82 ± 0.43	48.86 ± 0.64	48.76 ± 0.65	48.98 ± 0.53	0.46	0.89	0.27	0.84
Propionate	24.72 ± 0.33	24.52 ± 0.61	24.59 ± 0.19	24.59 ± 0.42	24.63 ± 0.43	24.39 ± 0.29	0.37	0.99	0.87	0.90
Butyrate	18.29 ± 0.36	18.90 ± 0.40	18.29 ± 0.62	18.75 ± 0.34	18.65 ± 0.81	18.54 ± 0.49	0.42	0.87	0.50	0.90
Isobutyrate	2.20 ± 0.24	2.15 ± 0.19	2.51 ± 0.32	2.19 ± 0.21	2.27 ± 0.20	2.34 ± 0.13	0.18	0.70	0.93	0.56
Isovalerate	3.21 ± 0.21	3.15 ± 0.37	3.35 ± 0.26	3.06 ± 0.30	3.17 ± 0.43	3.21 ± 0.26	0.22	0.95	0.97	0.86
Valerate	2.31 ± 0.11	2.73 ± 0.10	2.44 ± 0.12	2.55 ± 0.13	2.53 ± 0.10	2.53 ± 0.12	0.11	0.16	0.06	0.94
Acetic:propionic acid ratio	1.99 ± 0.04	1.99 ± 0.06	1.99 ± 0.02	1.99 ± 0.05	1.98 ± 0.03	2.01 ± 0.04	0.035	0.99	0.68	0.93

CTL, control treatment; NA1, naringin (1.5 g/kg DMI); NA2, naringin (3.0 g/kg DMI); CHI1, chitosan (1.5 g/kg DMI); CHI2, chitosan, (3.0 g/kg DMI); and mixture of CHI and NA (CHI-NA, 1.5 + 1.5 g/kg DMI). ^1^ Standard error of pH is expressed in H ions; SEM, standard error; surface response: L, linear contrast; Q, quadratic contrast.

**Table 7:** Effect of NA and CHI supplementation on enteric CH_4_ production in heifers.

Item	Treatments						SEM	*p*-Value	Contrast	
	CTL	NA1	NA2	CHI1	CHI2	CHI–NA			L	Q
**Methane production per day and yield**										
CH_4_ (g day^−1^)	153.0 ± 11.8	168.1 ± 11.2	153.0 ± 7.4	158.3 ± 13.0	161.0 ± 10.0	161.5 ± 14.0	10.21	0.14	0.20	0.70
CH_4_ (g kg^−1^ DMI)	18.09 ± 1.13	18.96 ± 0.78	18.04 ± 0.49	18.12 ± 0.60	18.98 ± 0.79	18.48 ± 1.14	0.69	0.57	0.60	0.95
**Methane g kg^−1^ per digestible fractions intake**										
CH_4_ (DM)	30.86 ± 2.25	33.87 ± 2.48	31.37 ± 1.85	32.15 ± 0.98	32.98 ± 2.01	32.27 ± 2.79	1.15	0.40	0.20	0.96
CH_4_ (OM)	35.43 ± 2.68	39.01 ± 2.97	35.91 ± 2.20	37.23 ± 1.44	37.75 ± 2.29	36.70 ± 3.49	1.46	0.49	0.20	0.98
CH_4_ (CP)	207.5 ± 12.64	230.2 ± 12.10	212.5 ± 7.20	225.7 ± 6.60	233.4 ± 10.08	218.9 ± 14.77	7.71	0.06	0.12	0.19
CH_4_ (NDF)	38.71 ± 2.51	43.38 ± 2.74	39.07 ± 1.71	41.25 ± 1.38	41.50 ± 1.93	39.86 ± 2.97	1.48	0.18	0.08	0.97
**Methane energy loss,** **Y_m,_ and emission factor**										
Energy loss as CH_4_ (MJ GEI day^−1^)	8.53 ± 0.66	9.35 ± 0.63	8.52 ± 0.41	8.81 ± 0.72	8.97 ± 0.56	8.99 ± 0.78	0.57	0.13	0.20	0.70
Y_m_ (% GE/day^−1^)	6.95 ± 0.43	7.28 ± 0.30	6.93 ± 0.19	6.96 ± 0.23	7.30 ± 0.30	7.11 ± 0.44	0.26	0.53	0.54	0.99
EF (CH_4_/head^−1^/year^−1^)	55.9 ± 4.30	61.3 ± 4.11	55.8 ± 2.71	57.7 ± 4.75	58.7 ± 3.67	58.9 ± 5.10	3.73	0.13	0.20	0.70

CTL, control treatment; NA1, naringin (1.5 g/kg DMI); NA2, naringin (3.0 g/kg DMI); CHI1, chitosan (1.5 g/kg DMI); CHI2, chitosan, (3.0 g/kg DMI); and mixture of CHI and NA (CHI-NA, 1.5 + 1.5 g/kg DMI); CH_4_: methane: CH_4_ day^−1^: CH_4_ g day^−1^; CH_4_DMI: CH_4_ g kg^−1^ dry matter intake; CH_4_DM: CH_4_ g kg^−1^ dry matter; CH_4_OM: CH_4_ g kg^−1^ organic matter; CH_4_PC: CH_4_ g kg^−1^ crude protein; CH_4_NDF: CH_4_ g kg^−1^ neutral detergent fiber; GEI: gross energy intake; Ym: CH_4_ MJ day^−1^, expressed as percentage of gross energy intake; EF: CH_4_ emission factor, kg CH_4_ head^−1^ year^−1^; SEM, standard error; surface response: L, linear contrast; Q, quadratic contrast.

## Data Availability

The data presented in this study are available on request from the corresponding authors.
